# Association of *CTLA-4 *gene polymorphisms with sporadic breast cancer in Chinese Han population

**DOI:** 10.1186/1471-2407-7-173

**Published:** 2007-09-10

**Authors:** Lihong Wang, Dalin Li, Zhenkun Fu, Heng Li, Wei Jiang, Dianjun Li

**Affiliations:** 1 Department of Immunology, Harbin Medical University, Harbin 150081, China; 2 Department of Surgery, the Third Affiliated Hospital of Harbin Medical University, Harbin 150081, China; 3 Department of Bioinformatics, Harbin Medical University, Harbin 150081, China

## Abstract

**Background:**

The host immunogenetic background plays an important role in the development of breast cancer. Cytotoxic T-lymphocyte antigen-4 (CTLA-4) is a molecule expressed predominantly on activated T cells and is important during the down-regulation of T-cell activation. To evaluate the potential influences of *CTLA-4 *gene polymorphisms on breast cancer risk, a case-control study was conducted in Han women of Northeast China.

**Methods:**

We genotyped *CTLA-4 *variants (-1661 G/A, -658 T/C, -318 T/C, +49 G/A and CT60 G/A) to tag all common haplotypes (≥ 1% frequency) in 117 Chinese breast cancer cases and 148 age/sex matched healthy individuals. Genotypes were determined by the polymerase chain reaction-restriction fragment length polymorphism (PCR-RFLP) method. Data was analyzed using the Chi-square test and Haploview software.

**Results:**

The frequency of *CTLA-4 *-1661G allele, -318T allele and CT60G allele carriers was significantly higher in patients than in controls (*P *= 0.0057, OR 1.91, 95% CI 1.21–3.02; *P *= 0.0031, OR 2.39, 95% CI 1.34–4.27; *P *= 0.023, OR 1.52, 95% CI 1.06–2.17, respectively). The -658T allele carrier frequency was significantly lower than in controls (*P *= 0.0000082, OR 0.17, 95% CI 0.08–0.37), whereas the +49A allele was significantly associated with tumor size in patients (*P *= 0.0033). Two common *CTLA-4 *haplotypes, ATCGA and ATCAG, were higher in healthy controls than patients (*P *= 0.0026, OR 0.17, 95% CI 0.05–0.54; *P *= 0.034, OR 0.12, 95% CI 0.02–0.92, respectively). A strong association was observed between tumor size and the ACCAA, ACCAG and ACCGA haplotypes (*P *= 0.0032, *P *= 0.0000031 and *P *= 0.017).

**Conclusion:**

These results suggest that polymorphisms of the *CTLA-4 *gene may modify individual susceptibility to and progression of breast cancer in Chinese Han women.

## Background

Breast cancer is the most common malignancy in women worldwide and its rate is increasing in both developed and developing countries. The etiology of breast cancer is complicated and not completely known, but recent studies have focused on the role of the immune system. During the development of breast cancer, innate and adaptive responses are carefully orchestrated through soluble and membrane-bound regulators, resulting in the deployment of the most suitable effectors for controlling the growth of tumor. However, the biological importance of these responses is not fully understood [1]. The most significant anti-tumor response is cell-mediated and involves T lymphocytes and natural killer (NK) cells. Thus, the variants of those genes that regulate the activation and proliferation of T lymphocytes and NK cells may affect the risk of breast cancer.

Cytotoxic T-lymphocyte antigen-4 (CTLA-4), encoded by a gene on chromosome 2q33, is a receptor expressed by activated T lymphocytes. It interacts with the B-7 cell surface molecule on antigen-presenting cells and inhibits T cell activation and clonal expansion [2,3]. CTLA-4 blockade leads to enhancement of the immune response [4], rejection of tumors [5], or even cures mice of tumors when used in combination with tumor vaccines [6]. The *CTLA-4 *gene consists of four exons and has been shown to be an important candidate gene for T cell-mediated diseases. More than 100 single-nucleotide polymorphisms (SNPs) have been identified in the *CTLA-4 *gene region [7]. Several important polymorphic sites have been reported in the entire region of the *CTLA-4 *gene. Among these variants, only A/G dimorphism at position +49 in exon 1 causes an amino acid exchange (threonine to alanine) in the peptide leader sequence of the CTLA-4 protein. Significantly increased expression of *CTLA-4 *mRNA and protein have been shown in individuals carrying thymine at position -318 of the *CTLA-4 *promoter and those homozygous for adenine at position 49 in exon 1 [8]. Ueda et al. reported that the G allele at the CT60 position was associated with a 50% decrease in the soluble CTLA-4 isoform [7]. Previous studies have extensively examined the association of polymorphisms within *CTLA-4 *with autoimmune diseases including Graves' disease, type I diabetes, systemic lupus erythematosus, and Hashimoto's thyroiditis [7,9-11], as well as malignancy susceptibility [12-15]. Some reports imply that the distribution of certain alleles of the *CTLA-4 *gene in cancer is contrary to autoimmune diseases: that is, those alleles that confer susceptibility to autoimmunity are sparse in patients with cancer or associated with good prognosis of the cancer. A recent report in an Iranian population showed that there was an association of an AA genotype at +49A/G with susceptibility to breast cancer and no significant differences in the promoter region (-1661 A/G and -318 C/T) of the *CTLA-4 *gene polymorphisms between patients and healthy controls [12,13]. To evaluate whether these polymorphisms of the *CTLA-4 *gene were likely to be of importance in Chinese breast cancer, we typed patients and controls for five of these important SNPs in the Chinese Han population of Northeast China. The five polymorphic sites of the *CTLA-4 *gene included three in the promoter region (-1661 G/A, -658 T/C, -318 T/C), one in exon 1 (+49 G/A) and one in 3'-UTR (CT60 G/A).

## Methods

### Subjects

The study group consisted of a total of 117 Chinese women with breast cancer, ranging in age from 30 to 73 years (mean age at diagnosis 48.7 ± 9.5 years). These patients came from the Department of Abdominal Surgery (The Third Affiliated Hospital of Harbin Medical University). Breast cancer was diagnosed from surgical and pathological symptoms. The patients' pathological and clinical information were obtained from their medical files (Table 1). The control group came from healthy blood donors consisting of 148 unrelated age (mean age at sampling 46.3 ± 17.0 years) matched women who were randomly selected from community volunteers in the same district, without a history of personal and familial malignancy and autoimmune disorders. Both patients and healthy controls originated from the Heilongjiang Province of China between September 2005 and June 2006. Samples were obtained from subjects after they had provided written informed consent.

**Table 1:** Clinicopathologic features of breast cancer patients (n = 117)

Feature	Cases, no. (%)
Tumor type	
IDC	111 (94.9)
MC	4 (3.4)
Other	2 (1.7)
Tumor size (cm)	
TZ≤2	39 (33.3)
2<TZ≤5	62 (53.0)
TZ>5	13 (11.1)
Unknown	3 (2.6)
LN involvement	
Positive	60 (51.3)
Negative	57 (48.7)

Abbreviations: IDC, infiltrative ductal carcinoma; MC, medullary carcinoma; LN, lymph node; TZ, tumor size.

### DNA extraction and genotyping

Genomic DNA was extracted from 5 ml frozen whole blood using the DNA Extraction Kit (Qiagen, Germany) according to the manufacturer's protocol. A polymerase chain reaction-restriction fragment length polymorphism (PCR-RFLP) assay was used to detect dimorphism of -1661 G/A, -658 T/C, -318 T/C, +49 G/A and CT60 G/A. The polymorphic region was amplified by PCR with a T-Gradient Thermoblock PCR System iometra, Germany) in a 25 μl reaction solution containing 0.3 μg genomic DNA, 1× PCR buffer, 0.3 mM MgCl_2_, 0.2 mM dNTPs, 2 U Taq DNA polymerase (Takara, Japan) and 0.1 μmol of each primer (Shenggong, China). SNPs were selected to tag all common (≥ 1% population frequency) *CTLA-4 *haplotypes. Genotyping primers and PCR programs are shown in Table 2. PCR products were digested overnight with restriction enzymes (NEB, UK) according to the manufacturer's protocol and analyzed by 2% agarose gel electrophoresis. Restriction enzymes and the length of digested fragments are shown in Table 3. This methodology was robust with a 97.8% genotype success rate [16].

**Table 2:** Primers and PCR programs for CTLA-4 PCR-RFLP genotyping

Reference SNP ID	Gene/SNP primer	Sequence	PCR program
rs4553808	*CTLA-4 *-1661G/A F	5'-CTAAGAGCATCCGCTTGCACCT-3'	94°C 5 min; 30 cycles, 94°C 15s,
	*CTLA-4 *-1661G/A R	5'-TTGGTGTGATGCACAGAAGCCTTT-3'	58°C 5s, 72°C 45s; 72°C 7 min
rs11571317	*CTLA-4 *-658T/C F	5'-ATCATTGGTCCTGTCTACAGC-3'	94°C 5 min; 35 cycles, 94°C 30s,
	*CTLA-4 *-658T/C R	5'-CTTCTAATGGTCCCTTGACAG-3'	55.5°C 10s, 72°C 30s; 72°C 5 min
rs5742909	*CTLA-4 *-318T/C F	5'-AAATGAATTGGACTGGATGGT-3'	94°C 4 min; 30 cycles, 94 °C 30s,
	*CTLA-4 *-318T/C R	5'-TTACGAGAAAGGAAGCCGTG-3'	55.6°C 5s, 72°C 30s; 72°C 4 min
rs231775	*CTLA-4 *+49G/A F	5'-AGTCTCACTCACCTTTGCAG-3'	94°C 4 min; 30 cycles, 94°C 30s,
	*CTLA-4 *+49G/A R	5'-GCTCTACCTCTTGAAGACCT-3'	56.4°C 10s, 72°C 30s; 72°C 4 min
rs3087243	*CTLA-4 *CT60G/A F	5'-GAGGTGAAGAACCTGTGTGTTAAA-3'	94°C 5 min; 30 cycles, 94°C 30s,
	*CTLA-4 *CT60G/A R	5'-ATAATGCTTCATGAGTCAGCTT-3'	55.6°C 30s, 72°C 30s; 72°C 5 min

**Table 3:** Restriction enzymes and length of digested fragments

	-1661G/A	-658T/C	-318T/C	+49G/A	CT60G/A
Enzyme	Mse I	Aci I	Mse I	Bbv I	HpyCH4 IV
Length of digested	A: 347+139	C: 254+112	T: 130+96+21	G: 255+56	G: 107+71
fragments (bp)	G: 486	T: 366	C: 226+21	A: 311	A: 178

### Statistical analysis

We tested for Hardy-Weinberg equilibrium (HWE) among cases and controls separately. SNPs were excluded from the analysis if they were out of HWE (*P *< 0.05), had a minor allele frequency of less than 5%, or had more than 25% missing data. The allele and genotype frequencies were obtained by direct counting. Statistical significance was defined as *P *< 0.05. We used Haploview 3.2 software to reconstruct haplotypes and estimate haplotype frequencies in the unrelated cases and controls. In order to obtain a measure of significance corrected for multiple testing bias we ran 10,000 permutations to compute *P-*values using the Haploview program. Comparisons of the distributions of the allele, genotype and haplotype frequencies were performed using the chi-square test. The relative risk associated with rare alleles was estimated as an odds ratio (OR) with a 95% confidence interval (CI).

## Results

### Genotype and allele frequencies

The genotype and allele frequencies of the *CTLA-4 *gene polymorphisms in breast cancer patients and healthy controls are shown in Tables 4 and 5. All of the five SNPs genotyped were in HWE (*P *> 0.05) in our case and control cohort. The minor allele frequencies were more than 5% and all of the missing data were less than 25%. When the genotype frequencies were compared between cases and controls, -1661, -658 and -318 SNPs showed statistically significant association (Details in Table 4). The *CTLA-4 *-1661 AA genotype and -318 CC genotype frequencies were lower in patients than in controls (*P *= 0.0022, OR 0.44, 95% CI 0.26–0.74; *P *= 0.0018, OR 0.37, 95% CI 0.20–0.69). The *CTLA-4 *-1661G allele, -318T allele and CT60G allele carrier frequencies were significantly higher in patients than in controls (*P *= 0.0057, OR 1.91, 95% CI 1.21–3.02; *P *= 0.0031, OR 2.39, 95% CI 1.34–4.27; *P *= 0.023, OR 1.52, 95% CI 1.06–2.17, respectively). In contrast, the -658 CC genotype frequency was significantly higher (*P *= 0.0000027, OR 6.92, 95% CI 3.08–15.52) (Table 4) and the T allele carrier frequency was significantly lower in patients than in controls (*P *= 0.0000082, OR 0.17, 95% CI 0.08–0.37) (Table 5). After highly conservative correction for multiple testing, significant association was only observed for the -1661G, -658T and -318T alleles (*P *= 0.031, 0.00010 and 0.017, respectively). Statistical analysis revealed no significant differences in the genotype and allele frequencies in +49 G/A between patients and healthy controls. Analysis of association between genotypes and predictive factors of breast cancer revealed no association between genotypes at these five SNP sites and lymph node metastasis. Tumor size was significantly different between carriers of the AA (*P *= 0.026) and GG (*P *= 0.0018) genotypes at position +49 G/A in comparison to other genotypes. In addition, we found a significant association between the +49A allele and tumor size (*P *= 0.0033).

**Table 4:** Genotype frequencies of CTLA-4 polymorphisms in breast cancer patients and healthy controls

Genotype		Frequency, no. (%)		*P*-value	Odds ratio (95% CI)
				
		Patients (n = 117)	Controls (n = 148)		
-1661, G/A^#^	AA	62 (56.9)	111 (75.0)	0.0022	0.44 (0.26–0.74)
	AG	45 (41.3)	35 (23.7)	0.0025	2.27 (1.33–3.87)
	GG	2 (1.8)	2 (1.4)	0.76	
-658, T/C^†^	CC	109 (94.8)	105 (72.4)	0.0000027	6.92 (3.08–15.52)
	TC	6 (5.2)	40 (27.6)	0.0000027	0.14 (0.06–0.32)
	TT	0	0		
-318, T/C	CC	84 (71.8)	129 (87.2)	0.0018	0.37 (0.20–0.69)
	TC	33 (28.2)	19 (12.8)	0.0018	2.67 (1.44–4.93)
	TT	0	0		
+49, G/A	AA	48 (41.0)	55 (37.2)	0.52	
	AG	59 (50.4)	70 (47.3)	0.61	
	GG	10 (8.6)	23 (15.5)	0.090	
CT60, G/A	AA	46 (39.3)	74 (50.0)	0.080	
	AG	47 (40.2)	56 (37.8)	0.70	
	GG	24 (20.5)	18 (12.2)	0.060	

^# ^patients (n = 109), missing (n = 8); controls (n = 148), missing (n = 0)

^† ^patients (n = 115), missing (n = 2); controls (n = 145), missing (n = 3)

**Table 5:** CTLA-4 allele counts and frequencies in breast cancer patients and healthy controls

SNP	Breast cancer (n = 117)		Healthy controls (n = 148)		*P*-value	Odds ratio (95% CI)
			
	Allele count (minor/major)	Allele frequency	Allele count (minor/major)	Allele frequency		
*CTLA-4 *-1661G/A^#^	49/169	22.5%	39/257	13.2%	0.0057 ^a^	1.91 (1.21–3.02)
*CTLA-4 *-658T/C^†^	6/224	2.6%	40/250	13.8%	0.0000082 ^b^	0.17 (0.08–0.37)
*CTLA-4 *-318T/C	33/201	14.1%	19/277	6.4%	0.0031 ^c^	2.39 (1.34–4.27)
*CTLA-4 *+49G/A	79/155	33.8%	116/180	39.2%	0.20	
*CTLA-4 *CT60G/A	95/139	40.6%	92/204	31.1%	0.023	1.52 (1.06–2.17)

^# ^patients (n = 109), missing (n = 8); controls (n = 148), missing (n = 0)

^† ^patients (n = 115), missing (n = 2); controls (n = 145), missing (n = 3)

^*a*^*P *= 0.031, ^*b*^*P *= 0.00010 and ^*c*^*P *= 0.017 after correcting the *P*-value for multiple testing by Haploview program using 10,000 permutations.

### Haplotypes analysis

Haplotypes with frequencies ≥ 1% are shown in Table 6. The most frequent haplotypes observed in patients and controls were haplotype ACCAG (-1661A, -658C, -318C, +49A, CT60G) (25.5%) and ACCGA (27.1%). However, statistical analysis did not reveal significant differences in the frequencies of these two haplotypes between patients and healthy controls. The frequencies of haplotypes, ATCGA and ATCAG, were significantly lower in patients compared with healthy controls (*P *= 0.0026 and *P *= 0.034, respectively). However, after the *P*-value was corrected for multiple testing using the permutation option available in Haploview 3.2 software, a significant association was only found for haplotype ATCGA (*P *= 0.012, OR 0.17, 95% CI 0.05–0.54).

**Table 6:** CTLA-4 haplotype frequencies in breast cancer patients and healthy controls

*CTLA-4 *haplotype					Freq.	Breast cancer (n = 117)	Healthy controls (n = 148)	*P*-value	Odds ratio (95% CI)
					
-1661	-658	-318	+49	CT60					
A	C	C	G	A	0.25	22.1%	27.1%	0.19	
A	C	C	A	G	0.22	25.5%	19.3%	0.089	
A	C	C	A	A	0.20	16.5%	22.5%	0.085	
G	C	T	A	A	0.062	8.1%	4.6%	0.081	
A	C	C	G	G	0.058	8.0%	4.1%	0.053	
G	C	C	A	A	0.052	6.5%	4.2%	0.22	
A	T	C	G	A	0.040	1.1%	6.3%	0.0026^a^	0.17(0.05–0.54)
G	C	C	A	G	0.033	3.9%	2.8%	0.50	
A	T	C	A	A	0.025	1.2%	3.5%	0.091	
G	C	T	A	G	0.016	2.2%	1.1%	0.32	
A	T	C	A	G	0.015	0.3%	2.5%	0.034	0.12(0.02–0.92)
A	C	T	A	A	0.010	2.0%	0.3%	0.062	

^*a*^*P *= 0.012 after correcting the *P*-value for multiple testing by Haploview program using 10,000 permutations.

Association with tumor prognostic or predictive factors was also observed with certain haplotypes. Although there was no association between haplotypes and lymph node metastasis in patients, we found strong association of tumor size with the ACCAA, ACCAG and ACCGA haplotypes (*P *= 0.0032, *P *= 0.0000031 and *P *= 0.017, respectively).

## Discussion

The determination of genetic polymorphisms is a new means to study the etiology of polygenetic disorders with complex inheritance patterns, such as cancer, diabetes and hypertension [17]. CTLA-4 exerts distinct independent effects during different phases of T cell responses, including setting the threshold for T cell activation, suppression of T cell proliferation, and induction of apoptosis in activated T cells [18]. It may even contribute directly to the regulation of B cell responses, since B cells express CTLA-4 after cell-cell contact with activated T cells [19]. Polymorphisms in the *CTLA-4 *gene have been tested for association with breast cancer in an Iranian population [12,13]. In the case-control study described here, we investigated the association between five putatively functional polymorphisms (-1661 G/A, -658 T/C, -318 T/C, +49 G/A and CT60 G/A) of the *CTLA-4 *gene and the risk of sporadic breast cancer in Chinese Han women.

A promoter region is the transcription factor binding site and regulates the level of expression of the gene [20]. This was the first study indicating that in the *CTLA-4 *promoter -658 CC genotype may increase breast cancer risk and -658T allele has a protective role in breast cancer. We also found that -1661 AA genotype and -318 CC genotype frequencies were lower in patients than in controls. The -1661G allele and -318T allele carrier frequencies were significantly higher in patients than in controls. These results suggest that these alleles might have an association with breast cancer risk in Chinese Han women. In contrast, there were no significant differences in genotype, allele, or haplotype frequencies in -1661 A/G and -318 C/T loci between patients and healthy controls in the Iranian population [13]. Some reports have suggested that the -318T allele may play a protective role in autoimmune disease [8,9]. The presence of the -318T allele may contribute to up regulation of *CTLA-4 *expression, and may therefore represent one mechanism that inhibits exaggerated immune activity [21]. Although we did not obtain the TT genotype of the -318 T/C site, our results show that T allele frequency was associated with an increased risk of breast cancer. Thus, our findings are consistent with earlier studies showing that the -318T allele increases the expression of *CTLA-4*, inhibits the activation of T lymphocytes and consequently limits the potency of tumor immunity. On the other hand, our results did not show an association of promoter genotypes and alleles with lymph node metastasis and tumor size at the time of diagnosis in breast cancer patients.

Among all of the *CTLA-4 *gene variants, the +49 G/A polymorphism is the most extensively studied, since this is the only polymorphism in the *CTLA-4 *gene that alters an amino acid [7]. Moreover, the *CTLA-4 *+49G allele has also been associated with incomplete glycosylation of the signal peptide and altered processing in the endoplasmic reticulum, which affects the CTLA-4-driven down regulation of T-cell activation and is an important factor in the pathogenesis of autoimmune diseases [16,22]. In our study, there were no significant differences in genotype or allele frequencies in +49 G/A between patients and healthy controls. However, the tumor size was significantly different between carriers of the AA and GG genotypes at position +49 G/A in comparison to other genotypes. This result suggests that +49 G/A was involved in the progression of breast cancer rather than in its initial development in Chinese Han women. The CT60 G/A polymorphism, located in 6.1-kb region 3' of the *CTLA-4 *gene, affects the ratio of trans-membrane to soluble mRNA splice forms of the *CTLA-4 *gene. Furthermore, the allele G is associated with lower levels of soluble CTLA-4 (sCTLA-4) expression. Genotypes producing higher levels of sCTLA-4 have been shown to protect against the development of autoimmune disease [23]. Many studies have reported that the CT60G allele was increased in patients of autoimmune diseases [24-26]. In our case-control study, we report for the first time that the CT60G allele shows a weak association with breast cancer, although the genotypic distributions were not significantly different between patients and controls. Our findings were in contrast to previous hypotheses proposing that alleles conferring susceptibility to autoimmunity are sparse in patients with cancer. To substantiate this result, further case-control studies of other ethnic groups are required. In addition, more studies should be carried out to clarify the exact mechanism of CT60 variant effects. In Taiwanese and Dutch populations, the *CTLA-4 *CT60G allele is the predominant one [24,27]. However, in our study, the CT60A allele is more prevalent than the allele G, consistent with previous reports [28]. Our study also revealed there was no association of CT60 genotypes or alleles with lymph node metastasis and tumor size at the time of diagnosis in breast cancer patients.

Previous studies have suggested that differences in CTLA-4 function are most likely to be associated with the haplotype rather than with individual SNP [29]. The frequencies of the haplotypes, ATCGA and ATCAG, were lower in patients compared with healthy controls. However, after the *P*-value was corrected for multiple testing only haplotype ATCGA showed a significant difference. This result might imply a protective role for these two haplotypes in combination of breast cancer in Chinese Han women. Tsung-Hsien Su et al. reported that CGG (-318C, +49G, CT60G) was the most prevalent haplotype in Taiwanese women [30]. In this context, the most frequent haplotypes in patients and controls were ACCAG and ACCGA. However, statistical analysis did not reveal significant differences in the frequencies of these two haplotypes between patients and healthy controls. Furthermore, we found significant association of ACCAA, ACCAG and ACCGA haplotypes with tumor size, suggesting that these three haplotypes participate in the progression of breast cancer.

## Conclusion

Our data suggest that the *CTLA-4 *gene may be involved in the susceptibility to and progression of breast cancer in the Chinese Han population. The promoter region (-1661 G/A, -658 T/C, -318 T/C) of the *CTLA-4 *gene may be the causal variants in breast cancer disease, whereas *CTLA-4 *+49 G/A may participate in the progression of breast cancer. In addition, this study also demonstrates that the *CTLA-4 *haplotypes, ATCGA and ATCAG, may have a protective role in breast cancer. We also show that three haplotypes, ACCAA, ACCAG and ACCGA, participate in the progression of breast cancer. Considering that the limited sample size may produce relative risk estimates lacking adequate precision, extended analyses with larger sample size should be carried out from different ethnic origins to further verify this association.

## Abbreviations

CI, confidence interval; CTLA-4, cytotoxic T-lymphocyte antigen-4; HWE, Hardy-Weinberg equilibrium; NK, natural killer; PCR-RFLP, polymerase chain reaction-restriction fragment length polymorphism; sCTLA-4, soluble CTLA-4; SNP, single nucleotide polymorphism

## Competing interests

The author(s) declare that they have no competing interests.

## Authors' contributions

Lihong Wang performed the primer design and wrote the drafts. Dalin Li collected the patient and control blood samples. Zhenkun Fu and Heng Li performed the PCR-RFLP experiments. Wei Jiang contributed statistical analysis. Dianjun Li conceived of the study, and participated in its design and coordination and helped to draft the manuscript. All authors read and approved the final manuscript.

## Pre-publication history

The pre-publication history for this paper can be accessed here:


